# Towards Understanding Early Failures Behavior during Device Burn-In: Broadband RF Monitoring of Atomistic Changes in Materials

**DOI:** 10.1149/2.0411609jss

**Published:** 2016-08-17

**Authors:** Yaw S. Obeng, Chukwudi A. Okoro, Papa K. Amoah, Johnny Dai, Victor H. Vartanian

**Affiliations:** a Engineering Physics Division, Physical Measurement Laboratory, National Institute of Standards and Technology, Gaithersburg, Maryland 20899, USA; b Theiss Research, La Jolla, California 92037, USA; c Department of Electrical Engineering, Frostburg State University, Frostburg, Maryland 21532-2303, USA; d Rudolph Technologies Inc., Bud Lake, New Jersey 07828, USA; e SEMATECH, Albany, New York 12203, USA

## Abstract

In this paper, we attempt to understand the physico-chemical changes that occur in devices during device “burn-in”. We discuss the use of low frequency dielectric spectroscopy to detect, characterize and monitor changes in electrical defects present in the dielectrics of through silicon vias (TSV) for three dimensional (3D) interconnected integrated circuit devices, as the devices are subjected to fluctuating thermal loads. The observed changes in the electrical characteristics of the interconnects were traceable to changes in the chemistry of the isolation dielectric used in the TSV construction. The observed changes provide phenomenological insights into the practice of burn-in. The data also suggest that these “chemical defects” inherent in the ‘as-manufactured’ products may be responsible for some of the unexplained early reliability failures observed in TSV enabled 3D devices.

The performance demands of emerging nanoelectronic devices appear to conflict with the need for reliability; they are expected to operate at higher current densities, with lower voltage tolerances and higher electric fields.^1^ These performance demands render new devices vulnerable to early failure. Thus, proper tradeoffs in the early design stage are now prerequisites for managing yields. This situation is further complicated by the introduction of new materials and integration schemes, and requires careful failure analyses to find new models for individual failure mechanisms and to identify possible interactions between them. This problem is further exacerbated by the fact that the mechanical and electrical properties of integrated circuits (IC) change during the fabrication process and over time, even in storage, due to the changes in chemistry of the materials of construction.^2,3^ Thus, there is a need for improved performance and material monitoring during and after device fabrication.

Classical models relate reliability to yield by assuming that devices are constructed out of perfectly stable materials, with well-defined and uniform physicochemical properties, and that the same extrinsic defects are responsible for limiting both die and packaged die yields. This implies that early reliability failures are attributable to defects present in the ‘as-fabricated’ devices.^1^ In reality, the reliability of electronic devices depends on the materials of construction, integration schemes, thermal history, use conditions, the structures of the devices, and test conditions.^2^ For example, several new failure mechanisms appear when devices are stressed at low voltages (1–3 V range), that are otherwise overshadowed by competing failures mechanisms when the systems are stressed at higher voltages and temperatures usually used for accelerated aging in reliability studies.^4^ Thus, there is a gap between the yield models and the physics of failure of IC devices. Hence, there is the need for effective reliability analyses to determine the dominant fundamental failure modes and their respective mechanisms (i.e., physics of failure), and to predict appropriate device lifetimes. We also need physical insights that would help us to understand, and validate, the assumptions entailed in reliability modeling, as well as test their sensitivities to the evolving materials and the integration schemes used in emerging nanoelectronics.

The reliability of electronic components is frequently described by the instantaneous failure rate or hazard rate, the time evolution of which resembles a ‘bathtub curve’. The ubiquitous bathtub curve represents the idea that the operation of a population of devices is comprised of three distinct periods: (1) an “early failure” (burn-in) period, where the failure rate decreases over time, (2) a “random failure” (useful life) period, where the failure rate is constant over time, and (3) a “wear-out” period, where the failure rate increases over time.^5^ The root cause of wear-out is the thermo-mechanical stresses that develop in the materials of construction and at material interfaces under fluctuating operating temperature profiles. The concept is frequently used in justifying burn-in strategies for improving system reliability.^3,6,7^ However, the value of the bathtub curve in characterizing infant mortalities has been questioned.^8^ The initial decreasing failure rates in the infant mortality region of the bathtub curve assumes some design or manufacturing defects cannot be completely eliminated, resulting in a subpopulation of “weak devices”; the “weak devices” could either die off completely or get stronger under stress. While this explanation of infant mortality is not analytically self-consistent, the bathtub model has historically been used successfully in the semiconductor industry.^8–10^ We need to develop physical evidence-based insights to help bridge the gap between theory and practice of burn-in, and to understand, at least phenomenologically, what happens during post device fabrication burn-in.

The chemistry and physics of materials play crucial roles in determining the reliability of nanoelectronic devices, especially in low voltage devices.^11^ Low-voltage devices are invariably put together using low temperature processes, such as plasma deposited (PECVD) and sub-atmospheric pressure (SACVD) deposited dielectric films derived from metallorganic precursors.^12^ Unfortunately, the low temperature processed materials are metastable from reliability perspectives due to the presence of reactive intermediates and incomplete reactions within the films. These inherent dangling / non-bridging bonds behave as electrical ‘defects’ and limit the utility of such materials in many possible applications.^13^ We recently reported on the changes in the electrical properties of the isolation dielectrics in through silicon vias (TSVs).^14^ The changes we observed were reminiscent of the changes in device performance during burn-in, where grossly defective devices are ‘weeded out’, while the recoverable defective “weak sisters” devices are ‘healed’.^15^ This apparent correlation motivated us to reexamine our TSV reliability historical data, and to relate the observed physicochemical changes in materials to the early failures and discuss them from the perspectives of the practice of ‘burn-in’ of integrated circuits. In this paper, we use our experimental data to propose an insight into the practice of device ‘burn in’ and to suggest that atomistic chemical transformation of inherent defects in the ‘as-manufactured’ products may be responsible for the success of the ‘bathtub’ modeling of infant mortality in semiconductor devices.^14^

## Experimental

For this study, SEMATECH (Albany, NY) fabricated TSV-enabled two-level stacked dies, bonded together with benzocyclobutene (BCB)^2^ were used. The TSVs were nominally 5.5 μm in diameter and 50 μm deep and were located at the top chip. The thicknesses of the isolation silicon-oxide liner (HARP, Applied Materials, Santa Clara, CA), and the barrier layer (TaN) were 500 nm and 25 nm, respectively. The details of fabrication are reported elsewhere.^16^

The samples were stored at room temperature in a nitrogen-purged enclosure for more than nine months before being used for this study. It is possible that the materials of sample construction (both metal and dielectric) may have undergone some room temperature annealing during storage. So, the observations reported in this work may not reflect all the potential changes possible in freshly fabricated devices. During thermal cycling, the samples were heated from 30°C to 150°C and then cooled to 30°C repeatedly in a nitrogen ambient. The thermal cycling was used to incrementally anneal and induce material changes in the devices.

For FTIR and other spectroscopic measurements, 500 ± 10 nm thick blanket silicon oxide films were deposited on 300 mm Si wafers, by sub-atmospheric pressure chemical vapor deposition from ozone and tetraethylorthosilicate gas mixtures (SACVD O_3_-TEOS, HARP, Applied Materials, Santa Clara, CA).^17^ The reported film thickness uniformity is one standard deviation (1−σ) from a 49-points thickness map, with 2 mm edge exclusion. To approximate the thermal history of the ‘as-received’ devices, the blanket wafers were first static annealed at 350°C for 10 minutes under nitrogen before being subjected the thermal cycling. For experimental convenience, we have chosen to focus on samples thermal cycled to 500 cycles in this paper.

Film properties such as thickness, density, modulus, roughness, and phase were measured with picosecond ultrasonic technology (PULSE) (Rudolph Technologies, Bud Lake, NJ).^18^ A pump-probe setup in which a 0.1 ps laser pulse (pump) is focused to about 5000 × 7000 nm^2^ spot onto a wafer surface to create an acoustic wave. The acoustic wave then travels away from the surface through the film. At the interface with another material, a portion of the acoustic wave is reflected to the surface while the rest is transmitted. When the reflected acoustic wave reaches the wafer surface, it is detected by another focused laser pulse (probe) which was diverted from the pump pulse by the beam splitter. Intrinsic physical properties of the film are calculated from first principles and do not require calibration standards or reference wafers.

For the dielectric spectroscopic studies, changes in the microwave scattering (S-parameters) were measured from 0.5 to 40 GHz using a PNA N5230A vector network analyzer (Keysight, Santa Clara, CA). Measurements were done at intervals of 500 thermal cycles. All measurements were made on devices without electrical bias. The transient insertion losses at the low frequency end of the broadband RF spectrum (below 300 MHz) were used to detect and characterize the response of device under test (DUT).

## Results

**Figure 1:**
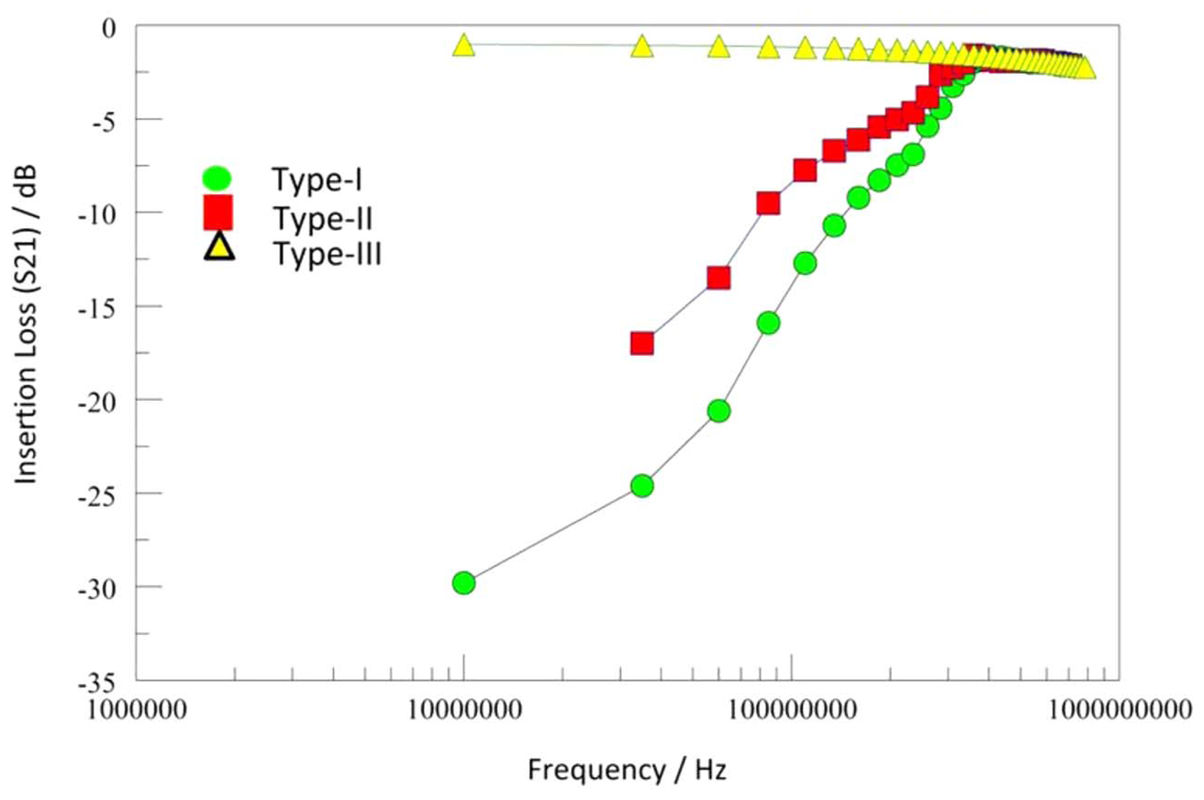
Comparison of the three defect types in the “as received” (zero thermal cycles) material. (Adpated from reference 14).

SACVD silicon-oxide films, such as O_3_-TEOS, are frequently used as the isolation liner dielectric in high aspect ratio features, such as in TSVs.^17^ However, the material reliability of such films are sub-optimal. For example, the time dependent dielectric breakdown (TDDB) lifetime of O_3_-TEOS based TSV isolation liner is far below ten years due to the transformation and degradation of the oxide material.^19^ The degradation byproducts of water and hydrogen suggest temperature and time driven chemical reactions involving possible condensation reactions of defects, such as silanol.^20^ The transformation of the dielectric material changes the electrical, and mechanical properties, of the TSV interconnects. We have previously reported on the detection and characterization of such defects in TSV enabled devices. Figure 1 suggests that there are at least three distinct classes of defects in the “as-manufactured” devices we studied. Interestingly, the defect Type-I converts to Type-II with modest thermal treatment. Ultimately, all the defects convert into the Type-III with time, although this step can be accelerated with temperature.^14^

Table I compares the characteristic relaxation time of the defects Types-I and –II respectively, with some benchmark values, for bulk pure water^21^ and adsorbed water on nonporous silica.^22^ The time constants observed in this work are about two orders of magnitude slower than those for the benchmarks, which is reasonable since the three systems are physically different from each other.^23^ In the present work we are looking at the non-bridging bonds grafted to the silicon oxide film, whereas Huang et al.^22^ looked at external water adsorbed on nano-porous silica matrices, and Kaatze^21^ looked at frozen pure water. In the condensed phase, water molecules are associated with each other through weak hydrogen bonds; the molecular rotation relaxation resonance frequencies (e.g., 20 GHz at 25C and 100 Hz at −80 C for crystalline ice) are indicative of the energy of coordination between the water molecules. In our study, the dangling bonds are not free to rotate, so the major relaxation frequencies are probably due to polarization induced “flip-flop” motions of the dangling bonds in the silicon oxide matrix in response to the rapidly changing electric fields of the microwaves. Thus, it is reasonable to observe slower relaxation of the dangling bond defects in the silicon oxide. Furthermore, a close inspection of Figure 1 reveals multiple slopes, suggesting that the signal loss traces may be actually composites of losses from a variety of defect types, which would be consistent with the wide range of polarizations associated with the multiple chemical environments and interfaces in such a complex matrix.^24^ In effect, the relaxation time constants for the defects in Table I represent the fastest of the species in each of the defect type categories.

**Figure 2:**
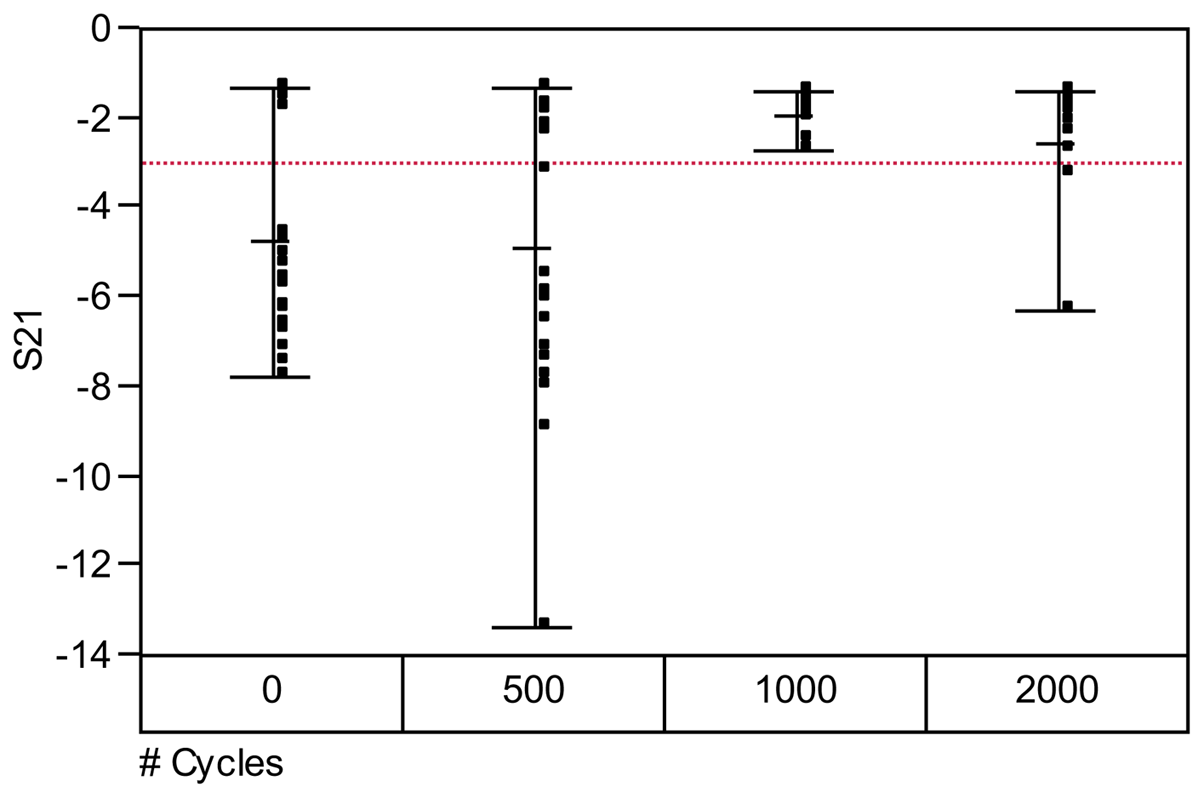
Distribution of Insertion losses (S21)) at 210 MHz as a function of number of thermal cycles. The horizontal dashed line denotes the −3dB (50% signal loss criterion).

**Figure 3:**
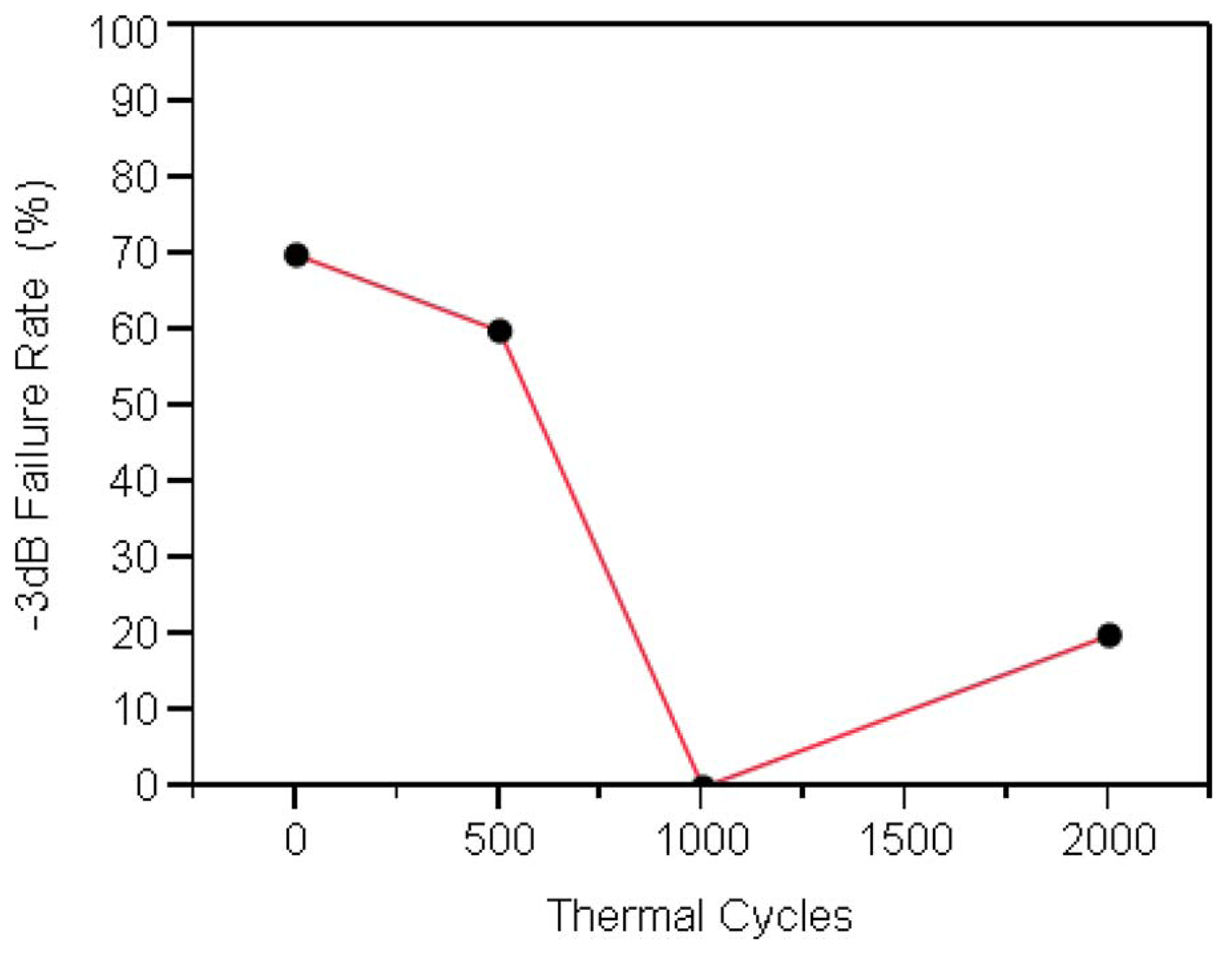
Fraction of Device Population Failing −3dB (50% Insertion losses (S21), at 210 MHz) criterion.

**Figure 4:**
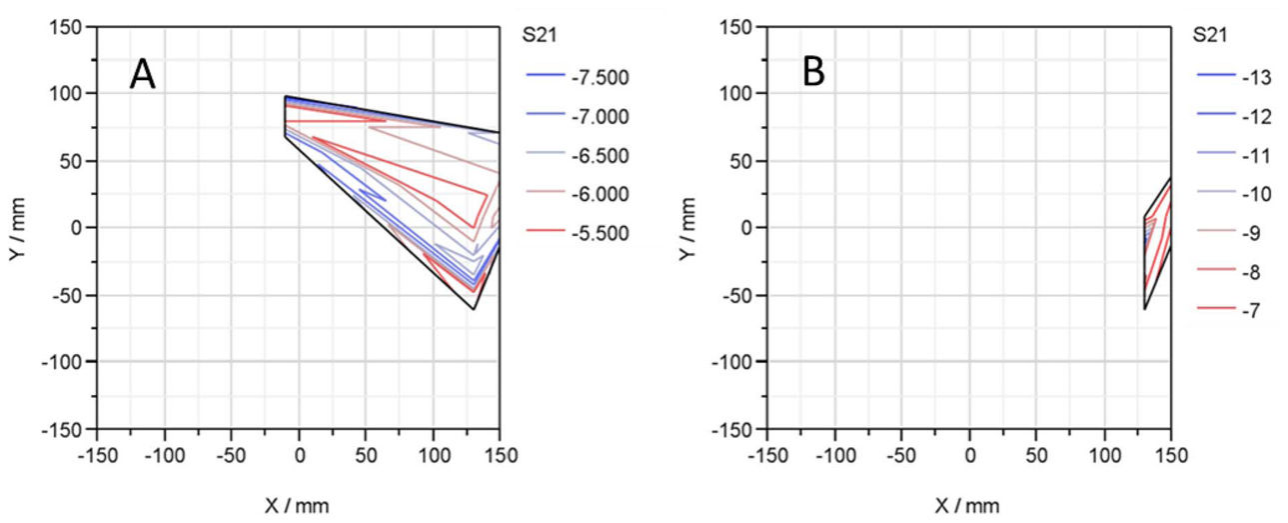
Comparison of the geographic distribution of failed dies in ‘as-received’ (A) and after 500 thermal cycles dies (B). The failing dies after 500 thermal cycles are concentrated in right edge of the wafer.

Figure 2 compares the microwave insertion loss (S21) magnitude, at 210 MHz, of a population of 30 ‘as received’ samples and subsequently thermally cycled (i.e., heat treated up to 2000 thermal cycles). In this report, we have arbitrary defined device failure as an insertion loss increase of 3dB, i.e., 50% loss of incident radiation at 210 MHz. Figure 3 shows the fraction of failing dies (expressed a failing rate) as a function of the number thermal cycles. As we have previously reported, the profiles of the S21 magnitude of the thermally cycled die population were much different from the ‘as-received’ population.^14^ The failure rate decreased monotonically with increasing number of thermal cycles, to a minimum at 1000 cycles, and then increased when cycled beyond that. The decreasing number of failing dies with thermal cycling is most likely related to atomistic transformations in the liner dielectric material with heat-treatment. We speculate that these reactions involve the conversion of silanol and silicon-ester defects into siloxane centers.^20,25^ Not all dies in the population studied were transformed into less lossy devices with the thermal cycling; the defects that could not be annealed out (i.e., hard-failed parts) continue to show very large RF signal losses even after extensive thermal treatment. Figure 4 compares the distribution of failed dies in ‘as-received’ and after 500 thermal cycles. Curiously, the “hard-failed” devices were concentrated in the right edge of the wafer. The concentration of “hard-failed” dies suggests that the chemical composition of the local dielectric differs substantially and did not undergo the same atomistic transformation that resulted in improved device performance elsewhere on the wafer.

**Figure 5:**
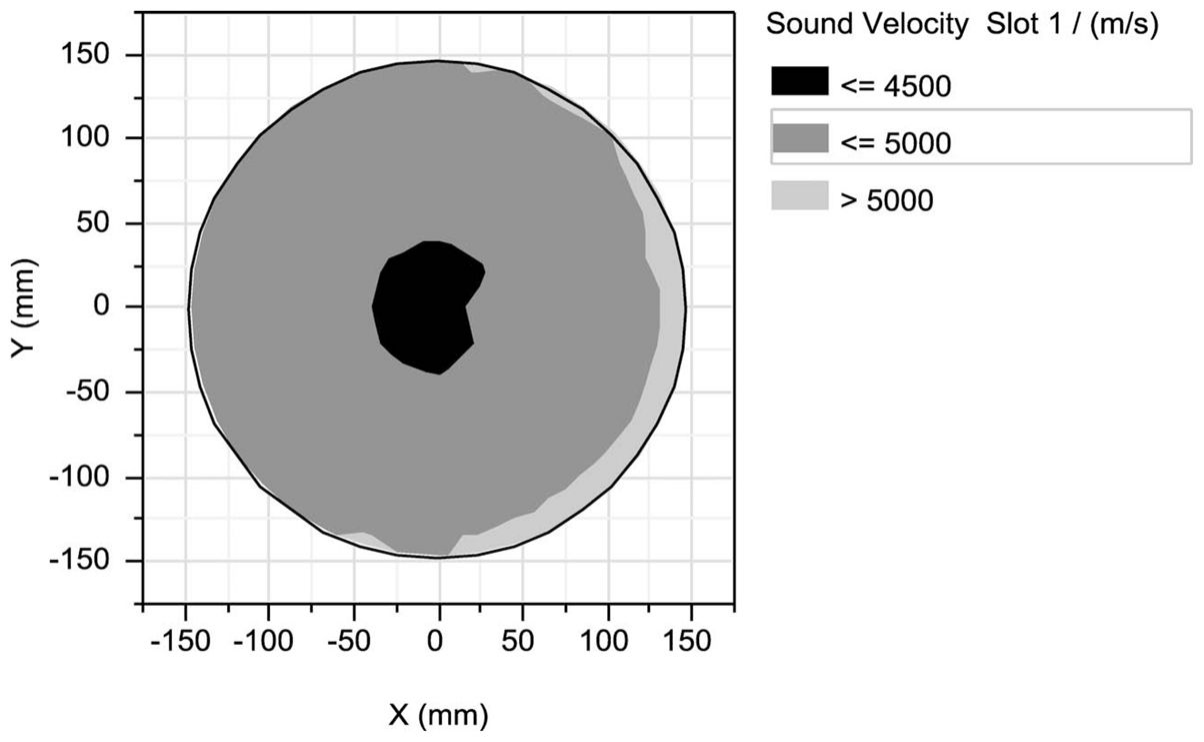
Segmentation of zones of constant sound velocity across a 500 ± 10 nm thick SACVD O_3_-TEOS film on a 300 mm silicon wafer.

**Figure 6:**
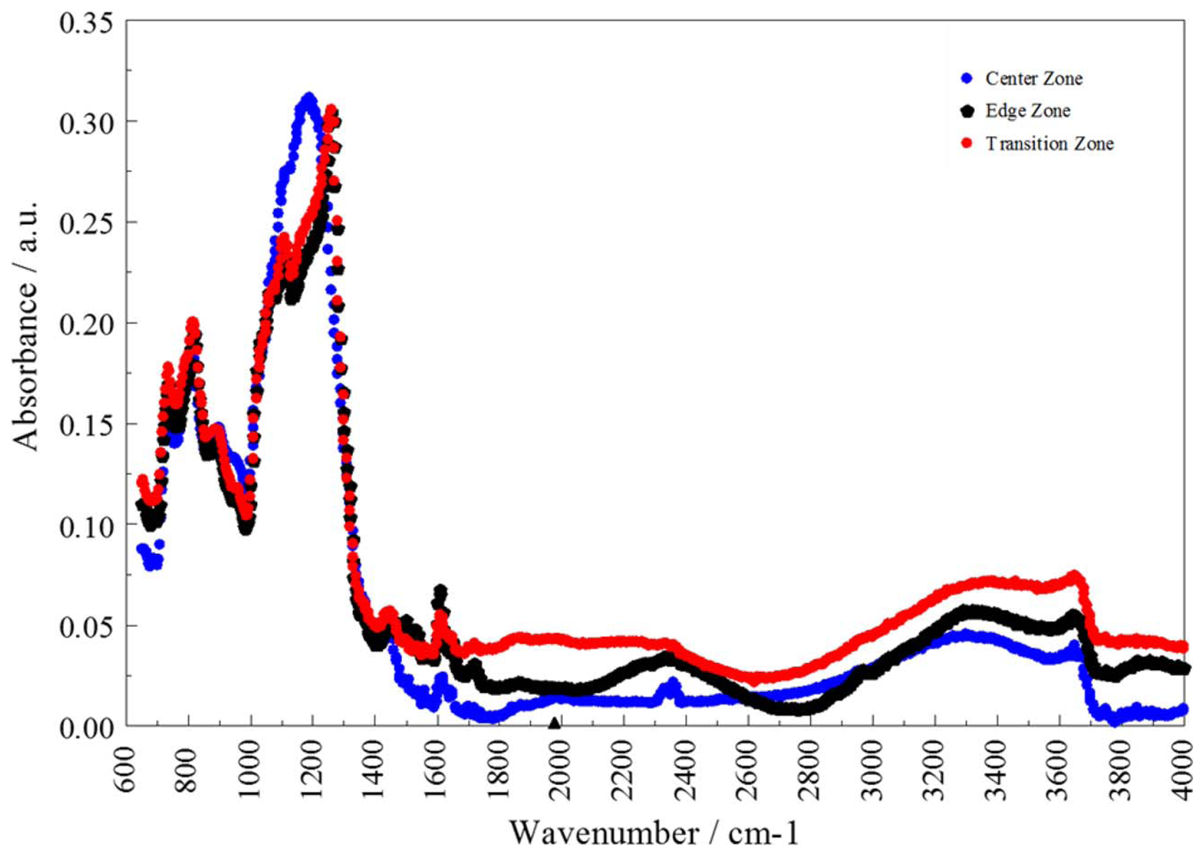
FTIR of blanket 500 nm SACVD O_3_-TEOS film after 500 thermal cycles between 30°C and 150°C, following a 10-minute static anneal at 350°C.

Indeed, as shown in Figure 5, acoustic impedance measurements on an ‘as-received’ 500 nm thick O_3_-TEOS film show three distinct zones of constant sound velocity. The edge band of acoustic impedance is much wider than the edge exclusion and one standard deviation of the thickness non-uniformity. As we have shown previously, the acoustic impedance, and other physical properties, of TEOS based thin oxide films depend on the local silanol concentration.^26^ Furthermore, as shown in Figure 6, the FTIR spectra after 500 thermal cycles suggest that quality and chemistry of materials from the right edge and the transition, are different from those from the center zone of Figure 5. While the former exhibit predominantly longitudinal optical (LO-mode) Si-O vibrations, the later exhibits predominantly transverse optical (TO-mode).^27^ This difference is attributable to the atomistic level structure of the silicon oxide, which depends on the local film deposition details, such as the local substrate temperature and or the composition of the reactant gases over the local area.^26,28^ Furthermore, all three zones show significant amounts of hydrogen –bonded silanol (~3650–3200 cm^−1^), as well as surface silanol (~3759 cm^−1^).^28^

**Figure 7:**
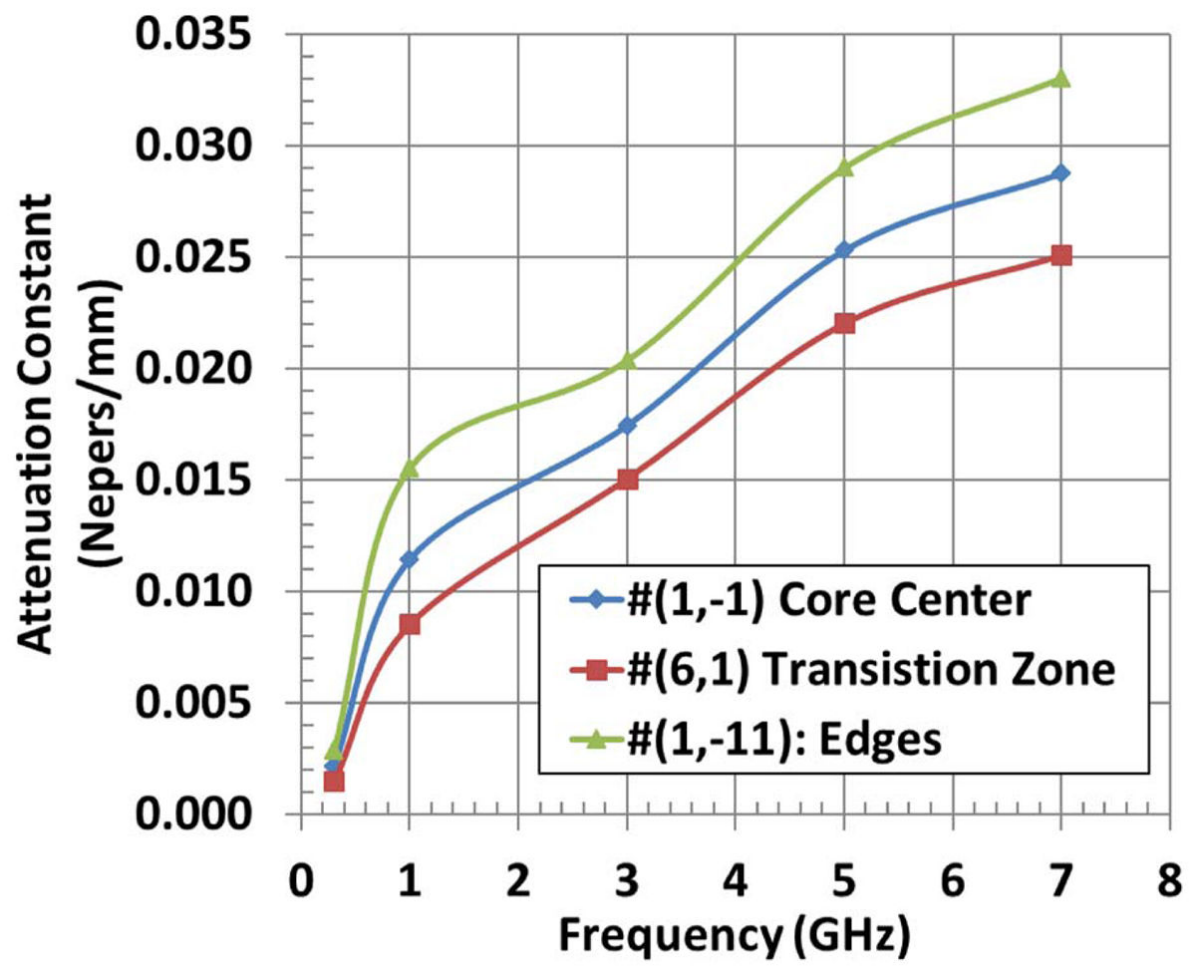
Comparison of the microwave attenuation constants in ‘as-manufactured’ devices selected from the three zones of constant sound velocity shown in Figure 5.

The microwave energy dissipation from scattering is accompanied by signal attenuation. Figure 7 is a comparison of the microwave attenuation constants^29,30^ in representative ‘as-manufactured’ devices selected from the three zones depicted in Figure 5. The data show distinct differences between the electrical performances of the devices from the center and the edge zones of the wafer. However, the performance of the devices form the transition zone was fluxional, spread around those from the two end zones.

## Discussion

The observations that the number of failing dies decreased monotonically with increasing number of thermal cycles, reaching a minimum at 1000 thermal cycles, suggests transformation in the materials of construction. For the simple devices studied in this work, this transformation could be in either the isolation dielectric or in the conducting metal fill of the vias. We believe the changes we describe here, at least at 500 thermal cycles, are more related to changes in the dielectric than to the metal fill, as TSV RF characteristics at low frequency (< 1 GHz), have been shown to be dictated by the capacitance of the isolation liner.^31^ However, we cannot totally discount contributions from the metal fill toward the improved device performance with thermal cycling.^32^ The changes in the device failure rate with thermal cycling is reminiscent of the changing characteristic product failure distribution with post-processing anneals and after product burn-in. It is assumed that during post-processing anneal and burn-in, the chemical bonds within a material strengthen over time, making their failure increasingly unlikely over time. In fact, the shape of Figure 3 fits a mathematical definition of the bathtub curve.^33^ The data presented in this work supports atomistic dielectric material transformation with thermal cycling as a contributor to the decreasing initial failure rate.

The RF insertion losses stem from polarization changes due to the reorientation of dangling bonds in the dielectric film. The partially ionic dangling bonds create interfacial polarizations which attempt to align their moment against the rapidly changing applied field, creating orientational polarization.^34^ This results in ‘flip-flop’ motions in the non-bridging bonds. The dipoles store energy when their moments are antiparallel to the applied electric field (ε′) and dissipate energy when they are out of phase with the applied electric field (ε″). The complex permittivity becomes frequency-dependent when dipoles can no longer stay in-phase with the applied field. At high frequency, dipoles do not get the opportunity to move much before the applied electric field changes orientation, and thus dipoles make insignificant contribution to ε′, whereas at low frequency, the dipoles are fully displaced, and their net moment stays antiparallel with the field, resulting in the maximum ε′. Between the high and low frequencies exists a relaxation frequency, where the dipoles are maximally out of phase with the applied field, resulting in peak ε″.^35^

In this work, dipolar relaxation govern the evolution of dielectric properties. Based on FTIR data, we ascribe the Type-I defects to the polarization silanols in the dielectric; and the changes observed after 500 thermal cycles to condensation reactions involving the silanols. When the devices are heated during the thermal cycling, the silanol non-bridging bonds presumably convert to siloxane bridging bonds, thus changing the electrical properties of the liner dielectric. These atomistic reactions remove of the dangling bonds, and thus eliminate the root causes of the energy loss, leading to reduced failure rates. We note that not all the dies in the population studied were transformed into less lossy devices with the thermal cycling, which suggests the dielectric material in the hard-failed area of the wafer did not undergo the same atomistic transformation that resulted in improved device performance.

The samples used in this work were stored at room temperature in a nitrogen-purged enclosure for more than nine months before use. It is possible that the materials of construction (both metal and dielectric) may have undergone some room temperature relaxation or annealing in storage. So, the observations reported in this work may not reflect all the potential changes possible in freshly fabricated devices. Room temperature anneal is improbable at least in the case of the copper fill and interconnect metallization. The Cu metallization in the ‘as-received’ test structures studied here had been stabilized during fabrication processing flow. The front- and back-side dielectric and etch stop depositions processes exposed the copper fill to heat-treatment of about 2 min at 350°C and 400°C, respectively.^32^ Furthermore, the completed devices were further annealed at 150°C for at least 1 h.^36^

The reliability changes we report in this paper could conceivably originate from other materials of construction beside the dielectric. For example, we,^32,37,38^ as well as others,^39^ have shown that the hydrostatic stress in typical Cu-filled SEMATECH fabricated TSV test structures change overtime, mostly due to changes in Cu grain size with additional post fabrication heating. We have also shown elsewhere that thermal cycling can lead to mechanical damage the SEMATECH fabricated test structures studied in this work.^40^ All these metal stress related changes are expected to be uniform across the wafer and do not show localization, and are different from the regional reliability issues reported in this paper. Besides the Cu, the only other materials in the test devices were dielectrics. As we discuss above the TSV isolation dielectric (SACD O_3_-TEOS) change with modest heating as non-bridging bonds condense into bridging bonds.^14^ Thus, it is reasonable to argue that the proposed atomistic transformation with thermal cycling that result in the decreasing initial failure rate, involves only to the TSV isolation dielectric material.

## Conclusions

We have applied a new way of detecting and characterizing different bond types and defects in dielectrics, based on changes in the low frequency (<300 MHz) dispersion of RF scattering, to gain insights into the changes that occur in O_3_-TEOS dielectrics during post processing heat-treatment. The utility of this method was demonstrated in characterizing the atomistic changes in SAVCD O_3_-TEOS used as an isolation liner in Cu TSVs fabrication during thermal stressing. In summary, the non-bridging / dangling bonds in the “as-processed” film react upon modest heat-treatment to form bridging bonds as a first step in the atomistic processes that result in improved reliability during device burn-in.

## Figures and Tables

**Table I:** Comparison of the characteristic relaxation time of the observed defect types in this work with some literature values obtained by terahertz spectroscopy on adsorbed water.

Species	Relaxation Time / s	Reference
Type-I	3.5 × 10^−10^	14
Type-II	4.3 × 10^−10^	14
Surface adsorbed water on nano-porous silica	4.8 × 10^−12^	22
Bulk pure water	8.3 × 10^−12^	21
